# Resuscitation plus – Initial successes and future direction

**DOI:** 10.1016/j.resplu.2022.100213

**Published:** 2022-02-17

**Authors:** Gavin D. Perkins, Janet Bray, Keith Couper, Peter Morley, Tommaso Scquizzato, Jerry P. Nolan

We are pleased to present this Editorial to the readership of Resuscitation Plus following the journal’s successful launch in 2020. Resuscitation Plus, is a companion title to the well-established journal Resuscitation.^1^ As the only fully open-access peer-reviewed journal dedicated solely to resuscitation, the journal aims to become the first choice for authors wishing to submit methodologically sound research relating to resuscitation in an open access journal. The journal’s scope includes a wide range of subject areas and paper types relevant to resuscitation (see Table 1).

**Table 1:** Resuscitation plus scope.

• Epidemiology of cardiac arrest • Prevention of cardiac arrest • Rapid response systems • Dispatch for cardiac arrest • Aetiology of cardiac arrest • Pathophysiology of cardiac arrest • Adult life support • Neonatal life support • Paediatric life support • Post resuscitation care • Cardiac arrest survivorship • Resuscitation training and simulation • Resuscitation in special circumstances (including trauma) • First aid topics	• Narrative, systematic and scoping reviews • Observation and randomised studies • Clinical and experimental studies • Qualitative studies • Methodology papers • Clinical trial protocols • Letters to the editor • Case reports

## Early success

At the time of going to press, Resuscitation Plus has accepted 188 articles from around the world (76 Europe, 70 North and Central America, 27 Asia, 13 Oceania, 2 Africa). The journal is pleased to have been indexed in the Directory of Open Access Journals, the Emerging Sources Citation Index, Scopus and PubMed Central. The journal is supported by an excellent editorial board of existing and emerging resuscitation leaders (see Table 2).

**Table 2:** Editorial board.

• Lars Andersen • Katherine Berg • Adam Cheng • Asger Granfeldt • Robert Greif • Taku Iwami • Andrew Lockey • Qingbian Ma • Ziad Nehme • Chika Nishiyama • Theresa Olasveengen • Yacov (Jack) Rabi • Giuseppe Ristagno • Jasmeet Soar

Articles published in the journal have been downloaded 170,635 times. The journals most downloaded articles include a randomised controlled manikin trial evaluating suction-based airway devices,^2^ a study of the impact of COVID-19 on out of hospital cardiac arrest in London,^3^ a dictionary for terms used to describe drowning and aquatic injuries,^4^ reviews relating to educational theories underpinning advanced life support courses^5^ and extracorporeal oxygenation,^6^ and the clinical trial protocol for a trial of vasopressin and methylprednisolone during in-hospital cardiac arrest.^7^ The journal has welcomed its first experimental medicine papers.8., 9. The importance of such papers for providing mechanistic insights and demonstrating proof of concepts for existing and future interventions for future clinical trials is highlighted in the editorial by Granfeldt et al.^10^

## International liaison committee on resuscitation

The journal is pleased to have established a collaboration with the International Liaison Committee on Resuscitation (ILCOR).^11^ ILCOR leads international efforts to continuously evaluate resuscitation research, seeking to develop consensus on science and treatment recommendations.^12^ Resuscitation Plus looks to support the evidence evaluation process by providing a repository to publish ILCOR systematic and scoping reviews. High quality reviews published to date have covered topics of willingness to perform bystander CPR,^13^ non-invasive monitoring during paediatric cardiopulmonary resuscitation,^14^ anticipatory manual defibrillator charging,^15^ CPR and defibrillation in the prone position,^16^ and briefing and debriefing in neonatal resuscitation.^17^

## European resuscitation council and young ERC

The European Resuscitation Council,^18^ the official sponsor of Resuscitation journal, have entered into a collaboration with Resuscitation Plus. The collaboration will have a particular focus on supporting the development of new researchers through the Young ERC group.^19^ Planned activities include appointing a Young ERC Editor and Editorial Board members. This will provide a developmental opportunity for a young researcher to learn about editorial processes, supported by the journal editorial team. With the Young ERC, we plan to develop a mentorship programme which will pair mentees with mentors to work together over a defined period with opportunities to publish in the journal.

## Social media

Social media is an important tool to further the reach and impact of scientific research. The journal has launched a Twitter profile to assist with dissemination. The PlumX tool indicates that articles published in the journal have been shared on Facebook or Twitter close to 4000 times. The recent appointment of Tommaso Scquizzato as social media editor will drive forward our communication via social media platforms. Follow the journal on Twitter at @Resus_Plus, retweet to your colleagues and peers, share your articles published in the journal, and join the conversation online with other experts in the fields of cardiac arrest and CPR.

## Future directions

Whilst it can take several years for a journal to receive an impact factor, we are optimistic the journal will receive one in the near future. Bibliometric scores of course provide just one lens for assessing a journal’s success. We prefer to judge the success of Resuscitation Plus through the quality of articles published and their impact on resuscitation practice and patient outcomes. With the journal’s desire to support the next generation of resuscitation scientists, we particularly welcome submissions from those early in their research careers. We hope the mentorship programme, being developed with the European Resuscitation Council will help accelerate that aspiration.

The journal wishes to extend its international research and we particularly welcome articles from low and middle income countries who may benefit from the Research4Life subsidy programme (www.research4life.org). The journal’s reviewers are critical to the journal’s success and we celebrate and acknowledge their contributions. As a small gesture of thanks, the journal is pleased to waive article processing fees for the journal’s most active reviewers.

As Resuscitation Plus continues on the journey to becoming the number one choice for researchers who wish to share methodologically sound, open access articles related to resuscitation, we look forward to the number and type of submissions continuing to grow.

## Declaration of competing interest

The editors receive support from Elsevier. All authors have volunteer roles with the International Liaison Committee on Resuscitation and their regional / national resuscitation councils.
